# Characterisation of Benzodiazepine Use in an Older Population Registered in Family Health Units in the Region of Minho, Portugal

**DOI:** 10.3390/geriatrics4010027

**Published:** 2019-03-01

**Authors:** Ana Vilaça, Ana Vieira, André Fernandes, Daniela Ribeiro, Inês Esteves

**Affiliations:** 1 Unidade de Cuidados de Saúde Personalizados Murtosa, 3870-264 Murtosa, Portugal; 2 Unidade de Saúde Familiar Manuel Rocha Peixoto, 4700-426 Braga, Portugal; amctvieira@arsnorte.min-saude.pt (A.V.); affernandes@arsnorte.min-saude.pt (A.F.); 3 Unidade de Saúde Familiar Lígios, 4750-062 Barcelos, Portugal; dfpribeiro@arsnorte.min-saude.pt; 4 Unidade de Saúde Familiar Cávado Saúde, 4750-511 Barcelos, Portugal; iesteves@arsnorte.min-saude.pt

**Keywords:** benzodiazepines, aged, aged 80 and over

## Abstract

Benzodiazepines are the most frequently consumed psychotropic drugs among older persons. This pharmacological class has been advised against in this group, due to the various risks associated with its use in an older population. This study seeks to determine the prevalence of benzodiazepine use in a non-institutionalized older population over the age of 75 that is registered in Family Health Units (USF) in the region of Minho, Portugal, as well as to characterize these patients and understand the link between benzodiazepine use and chronic medication use, risk of falls, and level of physical and functional dependence. The data extracted from the clinical records registered in the SAM^®^ were analyzed using the Statistical Package for the Social Sciences (SPSS). A sample of 700 patients was obtained. These patients presented a mean age of 82.3 years, 62.7% were female, 95.3% were physically independent, and 38.0% were functionally independent. Almost half of the elder persons presented a moderate (36.9%) or high (11.4%) risk of falls. 37.9% of the patients were chronic benzodiazepines users, using between 1 and 3 active substances belonging to this pharmacological class, with a higher rate of use among women (*p* < 0.001) and elder persons. There was a statistically significant association among the use of benzodiazepines, a functional independence, and a higher risk of falls. These pioneering findings in Portugal reveal a high prevalence of benzodiazepine use in the population studied and warn about the specific characteristics of said population and the importance in reducing the risks associated with the inappropriate prescription of these drugs.

## 1. Introduction

Benzodiazepines (BZD) are among the world’s most widely prescribed psychotropic drugs to older patients [1]. The use of these drugs among community-dwelling older people shows a large variability, ranging from 13.8% to 25.4% in international studies, depending on the used methodology [2,3,4,5,6]. These numbers are far greater among institutionalized patients [3].

Benzodiazepines are a potentially inappropriate medication in older people, due to the physiological changes associated with aging. Pharmacokinetic and pharmacodynamic alterations, comorbidities, and functional and social aspects inherent to aging make this population more vulnerable to its multiple adverse effects [1], namely, effects on postural control, higher risk of falls, fractures, dependence, cognitive changes and motor vehicle accidents, pain, depression and lower satisfaction with life, and greater difficulty in sleeping [6,7,8,9,10,11].

According to national and international standards and guidelines, benzodiazepine should not be used for longer than 12 weeks in any population [1,2]. This is particularly relevant in elderly patients, for whom, and according to the Beers and the STOPP/START criteria (tools that provide guidance for safe prescription in the elderly), the prescription is even discouraged.

Previous studies have shown that the chronic use of BZD in older patients is linked to several factors, namely: female gender, old age, multiple comorbidities, polymedication, overuse of healthcare services, low socioeconomic level, depressive symptoms, and institutionalization [1,11,12].

This study sought to determine the prevalence of benzodiazepine use in a non-institutionalized older population over the age of 75 that is registered in Family Health Units (USF) in the region of Minho, as well as to characterize these patients and understand the link between benzodiazepine use and chronic medication use, risk of falls, and level of physical and functional dependence.

## 2. Material and Methods

A retrospective, observational, transversal, and descriptive study was carried out in the Manuel Rocha Peixoto Family Health Unit (USF MRP). USF MRP is a small multi-professional team composed of 8 family doctors, 8 family health nurses, and 6 clinical technical assistants. It has organizational, functional and technical autonomy and provides individual and family health care services to a population of about 15,000 people from Braga. The target population consisted of patients over 75 years that were registered in this Family Health Unit. A representative sample of all patients over the age of 75 that were registered in the USF MRP in December 2013 was selected. Institutionalized patients, patients with no medical appointments in 2013, patients whose chronic medication had not been updated in the electronic prescribing programme, and deaths during the period in question were excluded.

During 2013, patients aged 75 years or more that were registered in the Manuel Rocha Peixoto Family Health Unit were scheduled medical and nursing appointments at the USF or at home. During these appointments, the chronic medication record of the patient was updated, and a set of scales was used to assess the risk of falls (Morse Scale), the physical dependence (Barthel Scale), and the functional dependence (Lawton & Brody Scale). In 2014, information related to the demographic profile (gender and age) of the patients was collected, as well as all the data that were gathered during the aforementioned appointments that took place in 2013, namely, data regarding the scales that measure the risk of falls and the physical and functional dependence, the number of chronically used drugs, the characterisation of BZD use (active substance and number of BZD), and the record of pathologies that might justify the prescription of these drugs.

The data were obtained by consulting the electronic clinical records registered in the SAM^®^. The collected information was logged and analyzed using SPSS (Statistical Package for the Social Sciences, Chicago, IL, USA; version 21.0). The numeric variables were described as mean, median, standard deviation, maximum and minimum value, for data with normal distribution. The categorical variables were described as absolute and relative frequencies (%) and, when relevant, with a confidence interval of 95% (CI 95%). The association between two independent categorical variables was assessed using Chi-square test (CS). The non-parametric Mann–Whitney test (MW) was used to compare a numeric variable between independent groups, because the normality assumption of the variables under study was always rejected. In order to confirm the normality of the variables, the Kolmogorov–Smirnov test was used. All statistical tests were two-sided, with a significance level of 0.05.

The present study was submitted to and approved by the Ethics Committee of the Administração Regional de Saúde do Norte (Regional Health Administration of the North) (Nº13/2015).

## 3. Results

### 3.1. Characterization of the Sample

From a total of 1043 patients aged 75 years or more, 343 were excluded for not fulfilling the inclusion criteria, thus resulting in a sample of 700 eligible patients for the study. These patients presented a mean age of 82.3 years (±4.9 years of age) and 62.7% (*n* = 439) were female. Each of these older patients had been prescribed a mean number of 5.9 (±3.1) drugs.

According to the Barthel Scale, out of a total of 507 patients, 95.3% (*n* = 483) were independent. According to the Lawton & Brody Scale, out of a total of 548 patients, 38.0% (*n* = 208) were functionally independent.

According to the Morse Scale, out of a total of 507 patients, 51.7% (*n* = 262) presented a low risk of falls, 36.9% (*n* = 187) presented a moderate risk of falls, and 11.4% (*n* = 58) presented a high risk of falls.

### 3.2. Characterisation of Benzodiazepine Use

Two hundred and sixty-five of the patients under study were chronic users of BZD, which represents a prevalence of 37.9% (CI 95% 34.3–4.5%) among older persons using BDZ for longer than 12 weeks. These patients presented a mean age of 82.6 years (±5.1 years of age) and 72.5% (*n* = 192) were female.

On average, BZD users used 1.08 (±0.32) active substances belonging to this pharmacological class, ranging between 1 and 3. Only 7.8% of these patients used more than one active substance. The most frequently prescribed active substances were lorazepam and alprazolam (Table 1).

After analyzing the clinical record of each patient, it was found that only 37.7% of the patients presented coded pathologies that potentially indicate the need for benzodiazepine use. Table 2 outlines the different pathologies registered in the clinical record that potentially justify the use of these drugs.

### 3.3. Characterisation of Benzodiazepine Users

Benzodiazepines are mainly used by women (43.7% versus 28.0%; *p* < 0.001) and are more frequently used by patients over the age of 85 years (43.2%) in comparison with patients in the 75 to 84 age group (35.4%), although this difference is in the threshold of statistical significance (*p* = 0.060).

Within the group of benzodiazepines users, the mean number of chronic medications was 7.1, a number statistically higher when compared to the mean number of 5.2 drugs prescribed to patients who do not use BZD (*p* < 0.001) (Table 3).

On the one hand, benzodiazepine use has been linked to a functional independence assessed by the Lawton & Brody Scale (*p* = 0.013), with its use being more common among independent patients (43.8%) than among dependent ones (33.2%). On the other hand, benzodiazepine use has shown an increased tendency to be more prevalent among patients who are physically dependent (54.2%), as evaluated by the Barthel Scale, than among those who are independent (37.3%), although this difference is not statistically significant (*p* = 0,096) (Table 3).

This study also found a correlation between an increase in the prevalence of benzodiazepine use and an increase in the risk of falls, with low-risk patients revealing a use of BZD of 32.8% versus a use of 50% among high-risk patients (*p* = 0.022) (Table 3).

## 4. Discussion

The prevalence of benzodiazepine use in a non-institutionalized older population over the age of 75 was 37.9%. This number is higher when compared to the results from the majority of the international studies previously carried out in similar populations [2,3,4,5,6]. The current prevalence among the Portuguese population is unknown. There is a Spanish study of an older community-dwelling population that showed a prevalence of 23.6% [1] and a more recent one that found that the use of anxiolytic and hypnotic drugs in a non-institutionalized older population was of 16.6% [12]. In this last study, the most frequently prescribed BZD was lorazepam (39.4% of the BZD), just like in our studied population.

A higher prevalence of benzodiazepine use among women is in agreement with previously published studies [2,12]. According to Fourrier et al., women are twice as likely to use BZD compared to men [2]. There are other factors that have been associated with the use of BZD, namely a low socioeconomic level, the presence of previous symptoms, an overuse of healthcare services, and polymedication [2,3,4,12]. The present study corroborates previously existing data by showing an association between polymedication and benzodiazepine use. There was no statistical significance in the association between aging and a prevalence of benzodiazepine use in the studied population, which can be justified by the old age of said population.

When assessing the reasons for prescribing benzodiazepine, by analyzing the codes used to identify the different pathologies, there is, most of the time, a considerable lack of potentially justifiable medical cause. According to a Swedish study, 57–58% of older patients chronically using BDZ presented a depressive and/or anxious disorder that led to the prescription of said drug. The absence of recorded pathologies that might justify the prescription of benzodiazepine may, however, be due to flaws in the codes and not to the lack of medical causes, a situation that must be clarified in future prospective studies. However, sleep disturbances assume a prominent role when it comes to prescribing these drugs, which should give cause for reflection within our community. A failure in the training of health professionals and of the general population regarding the characteristics of sleep in older people may justify this fact.

In this study, a significant association between functional independence and the use of BDZ was found, as well as a correlation (not statistically significant) between a higher use of these drugs and physical dependence. Although few in number, there are longitudinal studies that show that benzodiazepines are a significant predictor of the functional status in the older people, even though a causal connection has not yet been made [6]. Most of the research in this field portrays an association between the use of benzodiazepines and a physical and functional dependence [12,13]. In our studied population, there are various reasons that may justify these results, namely, the use of other hypnotic drugs in patients who present a higher functional dependence. This shall be researched in future studies.

There is also a known correlation between the use of benzodiazepines and their multiple adverse effects on older patients, namely, risk of falls, fractures of the femoral neck, depression, and cognitive changes [7,8,9,10,11,14]. The present study reveals that patients who do not use BZD present a lower risk of falls, which is in agreement with the literature. In fact, benzodiazepines are seen as a potentially inappropriate medication in the older due to their adverse effects, with an emphasis being given to falls because of the repercussions associated with them.

An understanding of geriatric medicine is important for undergraduates, postgraduate trainees in geriatric medicine, general practitioners, and allied health professionals [15]. The learning of geriatrics by doctors and nurses enables a reduction in the prescription of BZD as well as in all its impact on health. Further educational action regarding BZD prescriptions for older people which is referred to in the literature is: mailing evidence-based educational bulletins about BZD and also information sheets that physicians can give patients [16]; and the sharing of practical algorithms from updated international treatment guidelines among doctors to guide appropriate prescription of BDZs across different clinical scenarios and allow early detection of risk factors and potential indicators of misuse [17].

After this study, the USF MRP team took action at 3 levels: health care professionals, community, and BZD-consuming patients. They dedicated a week to the active and healthy aging, within which a day was created to allude to the use of BZD. They created a poster addressed to older people with sleep hygiene measures and BZD’s side effects. They wrote an article about the “World Sleep Day”, which was published in a local newspaper. They organized sessions addressed to older people and health care providers with an average and high risk of falls in which this potential adverse effect was approached. Additionally, a one-hour training session was provided to all the health care professionals about Beers and the STOPP/START criteria.

Although this study does bring forth extremely important scientific information regarding the community under study, it presents some limitations, such as possible registration omissions in the electronic clinical record, an inability to include sales without prescription, and the possibility of failure of patients to comply with prescribed medication regimens. Other weaknesses of this study include not only the absence of characterization of the type and dosage of benzodiazepine, but also of its prescription indications. In addition, the fact that the population under study is mainly female may lead to an association bias between the use of benzodiazepines and this gender. Finally, and although there is an increase in the prevalence of BZD use as well as an increase in the risk of falls, the association of the use of BZD with possible fractures was not investigated.

To conclude, the results show an alarming use of BZD among older patients. They also confirm that patients who do not use this drug have a lower risk of falls. Therefore, it is vital that physicians are made aware of this issue in order to minimize the risks associated with the inappropriate prescription of these drugs. It is essential—given the existence of a polymedicated population with high levels of BZD use—to introduce strategies for discontinuing benzodiazepine treatment in older patients and for a rational prescription in young adults.

## Figures and Tables

**Table 1:** Use of prescribed active substances among benzodiazepine users.

Active Substance	Prevalence (*n*)
Lorazepam	13.7% (96)
Alprazolam	9.0% (63)
Bromazepam	7.1% (50)
Diazepam	2.3% (16)
Cloxazolam	1.6% (11)
Mexazolam	1.3% (9)
Clorazepate dipotassium	1.3% (9)
Brotizolam	1.1% (8)
Other active substances	2.3% (16)

N.B.: The same patient might have been using more than one active substance.

**Table 2:** Prevalence of coded pathologies recorded in the clinical records of the patients that potentially indicated the need for benzodiazepine use.

Pathology	Prevalence (*n*)
P06-Sleep disturbance	5.0% (35)
P76-Depressive disorder	5.0% (35)
P70-Dementia	2.1% (15)
P01-Anxiety/nervousness/tension	1.9% (13)
P74-Anxiety disorder/anxiety state	1.6% (11)
P03-Feelings of depression	1.0% (7)
A04-Weakness/general tiredness	0.4% (3)
Other pathologies	2.6% (18)

N.B.: The same patient might have been suffering from more than one pathology.

**Table 3:** Association between benzodiazepine use and different variables.

	Does Not Use BZD (*n* = 435)	Uses BZD (*n* = 265)	*p*-Value
Barthel Scale, *n* (%) Dependent Independent	11 (45.8%) 303 (62.7%)	13 (54.2%) 180 (37.3%)	0.096
Lawton & Brody Scale, *n* (%) Dependent Independent	227 (66.8%) 117 (56.3%)	113 (33.2%) 91 (43.8%)	0.013
Number of drugs, mean (±SD)	5.2 (±2.9)	7.1 (±3.0)	<0.001
